# Case Report: Intercurrent infections in COVID-19-induced sustained immunodepression: is interferon gamma a suitable drug?

**DOI:** 10.3389/fimmu.2023.1183665

**Published:** 2023-06-08

**Authors:** Aurianne De Waard, Laurent Lefebvre, Julien Textoris, Didier Payen

**Affiliations:** ^1^ Intensive Care Unit, Centre Hospitalier Intercommunal Aix-Pertuis, Aix en Provence, France; ^2^ EA7426 “Pathophysiology of Injury-Induced Immunosuppression (PI3)”, Université Claude Bernard Lyon 1 - Hospices Civils de Lyon - bioMérieux, Lyon, France; ^3^ Université Paris 7 Denis Diderot, Unité de Formation et de Recherche (UFR) de Médecine, Paris, France

**Keywords:** COVID 19, interferon gamma (IFNγ), acute immuno-depression syndrome, ARDS (acute respiratory disease syndrome), HLA-DR

## Abstract

Acute immuno-depression syndrome (AIDs) had been observed in many life-threatening conditions leading to the Intensive Care Unit. and is associated with recurrent secondary infections. We report one COVID-19 patient with a severe ARDS, demonstrating acute immunodepression syndrome lasting for several weeks. The occurrence of secondary infections despite long treatment by antibiotics led to combined interferon γ (IFNγ) as reported previously. The response to IFNγ was evaluated by the flowcytometry HLA-DR expression on circulating monocytes, which was repeated from time to time. The severe COVID-19 patients responded well to IFNγ without adverse events.

## Introduction

Since January 2020, the COVID-19 outbreak continues to challenge the planet with multifaceted unresolved questions. Among these, the longitudinal immune response to SARS-COV2 associated with different clinical presentations from paucisymptomatic to severe acute respiratory distress syndrome (ARDS) is only partially understood. COVID-19 ARDS requires ICU hospitalization to support the failed lung function, ranging from supplementary oxygen delivery to lung assistance by venovenous ECMO technique (1). The systemic inflammation of these ARDS patients is associated in the early phase with an elevated plasma level of nonspecific markers (C Reactive Proteins, Ferritin, LDH, DDimers) (2), a profound reduction in the absolute number of peripheral total lymphocytes especially T4 and T8 cells having abnormal functionality (3), a normal or elevated peripheral monocyte absolute number, and a moderate increase in PMNs (3). The initial picture is moving along time in ICU concomitantly with the occurrence of secondary infections (4). Recent reports have shown an early downregulation of circulating mHLA-DR as a marker of acquired immunodepression syndrome (AIDs) (5). This syndrome had been reported in many ICU acute situations related or not to severe infection (6). High incidence of secondary infections seems to be real in ICU admitted COVID-19, questioning a potential role of a deep acquired immunodepression syndrome (AIDs) (7) with virus particle reactivation and frequent pulmonary infections with multi-resistant pathogens or not. In COVID-19 patients, this AIDs may relate to the reported inhibition of the IFN type I gene expression (8), the presence of auto-antibodies against IFNγ, (9) the low level of IFNγ release (10) and administration of therapies blocking an effective immune response (e.g. anti-IL-6, gluco-corticosteroids, JAK inhibitors). When intercurrent infections occur, the benefit of therapies boosting the host immunity has been tested in non-COVID (11), or in COVID-19 (12). We report here an ICU case of severe ARDS induced by an airway primary lung injury related to the SARS-Cov-2 infection. After several days, the patient had documented recurrent secondary infection, concomitant with documented AIDs. This immunodepression was successfully treated by IFNγ, which allowed indirect control of the infection by maintaining the same antimicrobial therapy, adapted to the documented bacterial sensitivity (11).

Case-1: A 60-year-old man with non-treated arterial hypertension and overweight (BMI =28.7kg/m²) was admitted to the ICU in March 2020 for an ARDS after a “flu-like syndrome” during the preceding two weeks. After 2 weeks of at-home prednisolone (20mg/day) treatment, his clinical status deteriorated with an arterial saturation at 70% requiring an emergency medical ambulance intervention. At arrival, the medical team decided to intubate and ventilate the patient before transportation to the ICU. At ICU admission, the examination showed: moderate tachycardia (90 bpm); elevated blood pressure (170/80 mmHg); moderate hyperthermia (38.0°C); and a 120 mmHg PaO_2_ at FiO_2_ 1 and PEEP 18 cmH_2_O. A positive PCR test for SARS-CoV-2 confirmed the COVID-19 diagnosis, highly suspected on a chest CT scan showing a major alveolo-interstitial pneumonia. The PCR tests were repeated 7 times during the ICU hospitalization, and it became negative after the day 25^th^ post-admission. The initial transthoracic 2D Echo-Doppler revealed a diastolic dysfunction with a hypertrophied left ventricle and an altered left ventricular ejection fraction (40%). The initial treatment consisted of hydroxychloroquine (200mg), ceftriaxone (2g), and azithromycin (500mg). The microbiological evaluation (pharyngeal swab) was positive for *Klebsiella pneumoniae* treated by cefepime for 8 days. Despite prone positioning and inhaled NO (10ppm; Inomax DS IR, Linde) the persistent severe hypoxia led to making the decision to start (day 4) a veno-venous ECMO assistance. Under methylprednisolone treatment (50mg q4 during 15 days), a distal lung-protected sampling at day 2 (+22 days after initial symptoms; 6^th^ day post-ICU admission) was positive *Enterobacter aerogenes* was treated by meropenem (2g q3), and rapidly replaced by piperacillin after testing the sensitivity.

**Figure 1:**
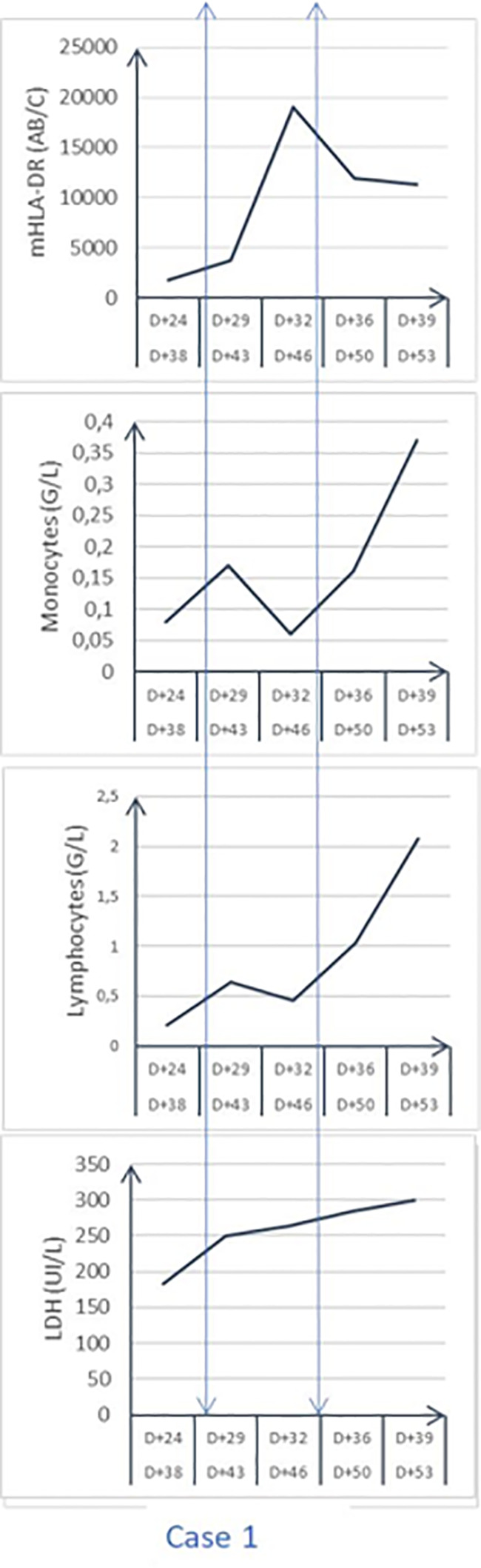
Time evolution of circulating monocyte HLA-DR expression (mHLA-DR; AB/number of events per cell), LDH, Lactico deshydrogenase enzyme; peripheral absolute cell count.

The improvement in lung function allowed us to wean the patient from the VV-ECMO after 11 days. On day 26 post ICU admission, bronchopneumonia was diagnosed with new images on the chest X-ray and gas exchange alteration. The distal lung-protected sampling was positive for wild-type *Pseudomonas aeruginosa* and *Enterobacter aerogenes* carrying a cephalosporinase, treated by piperacillin-tazobactam 4g q4, rapidly shifted after sensitivity testing to aerosols of colimycine, meropenem, and amikacine. The detected reactivation of CMV particles (RT-PCR) both in the lung and blood motivated to give ganciclovir for 2 weeks (10mg/kg/day). On the day 25 post-admission (32 days post symptoms) the laboratory exams showed a persistent absolute lymphopenia at 0.21G/L with a monocytopenia at 0.08 G/L, and a moderate pleocytosis (PMNs) with elevated ferritin (1238 ng/mL), LDH (250 UI/L), long-lasting positive SARS-Cov-2 PCR and severe clinical alteration. New investigations were then performed: - the myelogram that eliminated a macrophage activation syndrome; the repeated measurement of peripheral blood monocyte HLA-DR expression (flowcytometer FACSCanto II, Becton Dickinson, as previously reported) (13). The first mHLA-DR (day 24 after ICU admission) expression value was dramatically low (1760 AB/C), largely lower than the threshold diagnostic level of 8000 AB/C for AIDS (11). The diagnosis of AIDs associated with recurrent secondary infections, poor clinical status, and low absolute count of peripheral lymphocytes, motivated the decision to treat this AIDs at day 28 post-admission (day 42 post symptoms) with subcutaneous IFNγ (100mcg per injection; Immukin, Boerhinger, Germany), as previously reported (3). The IFNγ was chosen for several reasons: it has been tested safely many years ago and it is largely used for several immune diseases (14); it was shown efficient in severe ICU patients with AIDs (11) or severe fungal infections (15); - COVID-19 was shown to have a deficit in IFNγ release (9 16). After the agreement of the relatives for compassionate use, IFNγ was given at 100mcg per day subcutaneously for 7 days. Expression mHLA-DRwas monitored by flowcytometry monitoring (16) every 3 days. **Figure 1** shows the evolution of mHLA DR expression, lymphocyte absolute number having a peak at day 3 after IFNγ initiation. After stopping the drug administration, the mHLA-DR expression remained above the threshold of AIDs with no concomitant change in the absolute number of lymphocytes. As previously reported (3), lymphopenia improved after a long delay. The week after starting IFNγ treatment, the clinical status improved with no occurrence of a new secondary infection, allowing to wean the patient from mechanical ventilation. He was finally discharged from the ICU at day 54 after admission (68 days after initial symptoms).

## Discussion

This case demonstrates the possibility for a very severe COVID-19 patient may present an AIDs similar to the one observed in ARDS related to bacterial infection or non-infectious disease (17). As we have previously reported in longitudinal case series of sepsis with or without ARDS (3), occurrence of AIDs appears quite early, as an adaptive response to modulate the inflammatory response preventing tissue lesion induced by an inflammatory response (18). AIDs is not to be treated initially but monitored longitudinally to detect the persistence (stable low HLA-DR expression or trend to be lower) associated with the occurrence of secondary infections. In our previous report (11), the average delay between ICU admission and IFNγ administration was 18 days, a delay similar to the one observed in the present case (28 days). The failure to treat secondary infection despite adapted anti-microbial treatment suggests the necessity to boost innate immunity to synergize antibiotics and adequate innate immunity to control infection (19). The amplitude in mHLA-DR reduction, its duration, and the response to IFNγ seemed similar for this COVID-19 case in comparison with the one observed in the non-COVID patients (11). We and others have shown longitudinal results of the innate (mHLA-DR expression) and adaptive immunity (lymphopenia, reduced number and function of T4 and T8 cell in severe COVID-19 patients) (3, 20). The main information brought by this case report is to confirm that severe COVID-19 may induce a long-lasting AIDs, during which secondary infections may occur, as shown for other critically-ill patients as observed in other critically-ill patients (11, 21), The second important information concerns the ability of circulating monocytes to respond to IFNγ treatment. The third important result is to confirm that COVID-19 as in other ARDS etiology may require the combination of antimicrobial therapy and innate immune stimulation to resolve the secondary infection.

Among the potentially usable drugs to treat AIDs (19), the choice to use IFNγ was based on the role of interferon genes expression, the presence of auto-antibodies anti-INFγ (9), the low level of plasma INFγ, a relatively modest impact on lymphocyte number and function associated with an unclear impact of glucocorticoids. Moreover, IFNγ instead of IL-7 (22) or other drugs (23) is focused mainly on innate immunity, with a good tolerance and no-induced cytokine release and favorable kinetic effects (11). Lymphopenia with a reduced function has been shown (3, 24), and may contribute to impaired virus elimination observed in the present case. In the absence of immunomodulatory drugs, such a lymphopenia may last for several weeks even after IFNγ.

To summarize, severe COVID-19 may induce AIDs soon after ICU admission, which may persist for several days concomitantly with secondary infections as during other contexts of ARDS. In the absence of contraindication as macrophage activating syndrome, IFNγ is efficient to recover innate immune function and to clear viruses and bacterial infections.

## Data availability statement

The original contributions presented in the study are included in the article/supplementary material Further inquiries can be directed to the corresponding authors.

## Ethics statement

Ethical review and approval was not required for the study on human participants in accordance with the local legislation and institutional requirements. The patients/participants provided their written informed consent to participate in this study. Written informed consent was obtained for the publication of this case report.

## Author contributions

All authors contributed to the article and approved the submitted version.
